# Immunogenicity and safety of the 2015 Southern Hemisphere formulation of a split-virion inactivated quadrivalent vaccine

**DOI:** 10.1080/21645515.2017.1377378

**Published:** 2017-10-30

**Authors:** Cecilia Montalban, May Book Montellano, Jaime Santos, Nathalie Lavis

**Affiliations:** a Manila Doctors Hospital, Infectious Disease Section, Department of Medicine, Ermita, Manila, Philippines; b Mary Chiles General Hospital, Department of Pediatrics, Sampaloc, Manila, Philippines; c Philippine Children's Medical Center, Infectious Diseases Section, Quezon, Quezon City, Philippines; d Sanofi Pasteur, Medical Operations, Lyon, France

**Keywords:** clinical trial, immunogenicity, quadrivalent inactivated influenza vaccine, safety, Southern Hemisphere

## Abstract

An inactivated split-virion quadrivalent influenza vaccine (IIV4; Fluzone® Quadrivalent; Sanofi Pasteur) has been available in the US since 2013 and in the Southern Hemisphere since 2015. Here, we describe the results of an open-label, post-licensure trial (WHO Universal Trial Number, U1111-1143-9256) to confirm the immunogenicity and safety of the Southern Hemisphere 2015 formulation of IIV4. Adults 18–60 years of age and > 60 years of age (n = 60 per age group) received a single 0.5-mL intramuscular injection of IIV4. After vaccination, hemagglutination inhibition titers for each strain in IIV4 increased by a geometric mean of at least 10-fold for younger adults and at least 9-fold for older adults. All of the younger adult participants and 98%–100% of the older adult participants had seroprotective titers for each strain. Also, at least 80% of younger adults and 78% of older adults seroconverted or had a significant increase in titer for all four vaccine strains. These post-vaccination immune responses exceeded the criteria of the Committee for Human Medicinal Products former Note for Guidance for influenza vaccines. Finally, no serious adverse events were reported, and no new safety signals were detected. These results confirmed that the Southern Hemisphere 2015 formulation of IIV4 was well tolerated, highly immunogenic, and met the criteria for influenza vaccine immunogenicity and safety.

A quadrivalent inactivated split-virion influenza vaccine (IIV4; Fluzone® Quadrivalent, Sanofi Pasteur) has been available in the US since 2013 and in the Southern Hemisphere since 2015 for individuals ≥ 6 months of age.^1^ IIV4 contains hemagglutinin from two influenza A strains (H1N1 and H3N2) and both B-strain lineages (Victoria and Yamagata), in contrast to trivalent vaccines, which contain only a single B-strain lineage. IIV4 builds on the extensive experience with the trivalent split-virion influenza vaccine (IIV3; Fluzone®, Sanofi Pasteur), which has been available since 1980.

The immunogenicity and safety of IIV4 were examined in a phase II trial in adults ≥ 18 years of age^2^ and a phase III trial in children 6 months to 8 years of age.^3^ In both studies, participants were randomized to be vaccinated with IIV4, a IIV3 containing the B Yamagata-lineage strain, or a IIV3 containing the B Victoria-lineage strain. Both studies showed that IIV4 induced hemagglutination inhibition (HAI) antibody titers that were statistically non-inferior to those induced by the IIV3s for the two A strains and non-inferior for the B-strain lineage when present in the IIV3 comparator. For the B-strain lineage not present in the IIV3 comparator, IIV4 induced HAI antibody titers that were statistically superior. Incidences and severities of solicited reactions and unsolicited adverse events in these studies did not differ between IIV4 and the IIV3s.

Following approval of IIV4 in the Southern Hemisphere in 2015, the Brazilian health authorities (*Agência Nacional de Vigilância Sanitária*) requested that the immunogenicity and safety of the 2015 formulation be confirmed according to the criteria of the Committee for Human Medicinal Products (CHMP) former Note for Guidance on influenza vaccines.^4^ Therefore, in an open-label, post-licensure trial conducted in the Philippines (WHO Universal Trial Number, U1111-1143-9256), 60 younger adults (18–60 y) and 60 older adults (> 60 y) not vaccinated for seasonal influenza in the previous 12 months received a single dose of IIV4. Details of the exclusion criteria and study ethics are provided in the Supplemental Online Information. Most of the participants in both age groups were female (25 men and 35 women in the younger adult group and 15 men and 45 women in the older adult group), and all completed the study.

HAI antibody titers were measured at baseline (day 0) and 21 days (window, 21–28 days) after vaccination in all vaccinated subjects with data available, as described previously^2^ (see Supplemental Online Information for further detail). Before vaccination, between 43.3% and 95.0% of the participants in each age group had seroprotective HAI antibody titers (≥ 40) against each of the vaccine, and baseline titers were relatively high (Table 1). This likely reflects earlier exposure to influenza viruses through natural infection or previous successive influenza vaccinations, although none of the participants reported having had influenza or a seasonal influenza infection during the previous year. Despite these relatively high baseline titers, HAI titers for each strain in IIV4 increased after vaccination by a geometric mean of at least 10-fold for younger adults and at least 9-fold for older adults. At least 80% of younger adults and 78% of older adults seroconverted or had a significant increase in titer for all four vaccine strains. Finally, all of the younger adult participants and 98%–100% of the older adult participants had seroprotective titers for each strain after vaccination. For both age groups, these post-vaccination immune responses for all strains met all of the former CHMP criteria, although seroprotection is no longer used in the updated CHMP guidelines that became available in 2016^5^ and is losing favor as an estimate of protection.^6-9^

**Table 1.** Immunogenicity.

				A/H1N1	A/H3N2	B Yamagata	B Victoria
Age group	Measure	Day	EMA criteria^a^	N = 60	N = 60	N = 60	N = 60
18–60 y	HAI GMT^b^	0	–	74.6 (49.9, 112)	27.8 (19.3, 39.9)	209 (154, 283)	80.0 (58.6, 109)
		21	–	2009 (1648, 2448)	532 (390, 726)	2242 (1828, 2749)	1052 (849, 1303)
	Seroprotection^c^, %	0	–	68.3 (55.0, 79.7)	43.3 (30.6, 56.8)	95.0 (86.1, 99.0)	80.0 (67.7, 89.2)
		21	> 70%	100.0 (94.0, 100.0)	100.0 (94.0, 100.0)	100.0 (94.0, 100.0)	100.0 (94.0, 100.0)
	GMTR^d^	21/0	> 2.5	26.9 (17.1, 42.4)	19.1 (12.5, 29.4)	10.7 (7.95, 14.5)	13.1 (9.72, 17.8)
	Seroconversion/significant increase^e^, %	21/0	> 40%	86.7 (75.4, 94.1)	80.0 (67.7, 89.2)	85.0 (73.4, 92.9)	90.0 (79.5, 96.2)
> 60 y	HAI GMT^b^	0	–	48.4 (33.2, 70.5)	34.4 (24.6, 48.1)	118 (86.0, 161)	44.9 (32.3, 62.4)
		21	–	1083 (813, 1442)	723 (525, 994)	1070 (835, 1371)	835 (654, 1066)
	Seroprotection^c^, %	0	–	61.7 (48.2, 73.9)	45 (32.1, 58.4)	81.7 (69.6, 90.5)	61.7 (48.2, 73.9)
		21	> 60%	100.0 (94.0, 100.0)	98.3 (91.1, 100.0)	100.0 (94.0, 100.0)	100.0 (94.0, 100.0)
	GMTR^d^	21/0	> 2	22.4 (14.7, 34.0)	21.0 (14.0, 31.5)	9.08 (6.49, 12.7)	18.6 (13.6, 25.4)
	Seroconversion/significant increase^e^, %	21/0	> 30%	86.7 (75.4, 94.1)	86.7 (75.4, 94.1)	78.3 (65.8, 87.9)	91.7 (81.6, 97.2)

This was an open-label trial (WHO Universal Trial Number, U1111-1143-9256) conducted at three sites in the Philippines between June 17 and July 17, 2015. The study included 60 healthy younger adults (18–60 years of age) and 60 healthy older adults (> 60 years of age) not vaccinated for seasonal influenza within the previous 12 months. All participants received a single 0.5-mL injection of the 2015 Southern Hemisphere formulation of quadrivalent split-virion influenza vaccine (Fluzone® Quadrivalent, Sanofi Pasteur). Each 0.5-mL dose contained 15 μg of hemagglutinin per strain of A/California/07/2009 (H1N1), A/South Australia/55/2014 (H3N2), B/Phuket/3073/2013 (Yamagata lineage), B/Brisbane/60/2008 (Victoria lineage). Further details of the exclusion criteria and study ethics are provided in the Supplemental Online Information. Blood was collected before vaccination (day 0) and 21 days (window, 21–28 days) after vaccination. Serum HAI titers were measured in all vaccinated subjects with data available and as described previously.^10^ HAI titers under the lower limit of quantitation (10) were assigned a value of 5, and all HAI titers above the upper limit of quantitation (10,240) were assigned a value of 10,240. Data were analyzed using SAS® version 9.4 (SAS Institute). Values in brackets indicate 95% CIs. Abbreviations: CI, confidence interval; EMA, European Medicines Agency; GMT, geometric mean titer; GMTR, geometric mean titer ratio; HAI, hemagglutination inhibition;

a EMA Committee for Human Medicinal Products former Note for Guidance on Harmonisation of Requirements for Influenza Vaccines (CPMP/BWP/214/96).^4^

b GMTs and 95% CIs were determined from log_10_-transformed data using Student's *t*-distribution with n−1 degrees of freedom, after which antilog transformations were applied to the results of calculations.

c Seroprotection was defined as a HAI titer ≥ 40.

d GMTR was calculated as the geometric mean of the individual ratios of the post-vaccination (day 21) HAI titer divided by the pre-vaccination (day 0) HAI titer. GMTRs and 95% CIs were determined from log_10_-transformed data using Student's *t*-distribution with n−1 degrees of freedom, after which antilog transformations were applied to the results of calculations.

e Seroconversion was defined as a pre-vaccination (day 0) HAI titer < 10 and a post-vaccination (day 21) HAI titer ≥ 40. A significant increase was defined as a pre-vaccination HAI titer ≥ 10 and a ≥ 4-fold post-vaccination increase in HAI titer.

As found previously in adults,^2^ injection-site pain, headache, malaise, and myalgia were the most frequently reported solicited reactions to vaccination (Table 2). All injection-site and most systemic reactions were grade 1 or grade 2 severity, and all resolved within 7 days. The only grade 3 reactions were two cases of fever: one was reported by a younger adult and resolved spontaneously within 5 days, and the other was reported by an older adult and resolved with medication within 1 day. CHMP-defined reactions reported within 3 days after vaccination included malaise (11.7%) and shivering (3.3%) in younger adults and malaise (3.3%), shivering (1.7%), and temperature > 38.0°C lasting for at least 1 day (1.7%) in older adults. No immediate unsolicited adverse events (within 30 min), vaccine-related unsolicited adverse events, serious adverse events, or adverse events leading to study discontinuation were reported.

**Table 2.** Unsolicited AEs and solicited reactions.

	18–60 y		> 60 y	
	N = 60		N = 60	
Reaction	n	% (95% CI)	n	% (95% CI)
Unsolicited AEs during the study^a^	7	11.7 (4.8, 22.6)	5	8.3 (2.8, 18.4)
Immediate events (< 30 min)	0	0.0 (0.0, 6.0)	0	0.0 (0.0, 6.0)
Vaccine-related events	0	0.0 (0.0, 6.0)	0	0.0 (0.0, 6.0)
Leading to study discontinuation	0	0.0 (0.0, 6.0)	0	0.0 (0.0, 6.0)
Serious adverse events	0	0.0 (0.0, 6.0)	0	0.0 (0.0, 6.0)
Injection-site reaction within 7 days	19	31.7 (20.3, 45.0)	14	23.3 (13.4, 36.0)
Pain	19	31.7 (20.3, 45.0)	14	23.3 (13.4, 36.0)
Erythema	0	0.0 (0.0, 6.0)	0	0.0 (0.0, 6.0)
Swelling	0	0.0 (0.0, 6.0)	1	1.7 (0.0, 8.9)
Induration	1	1.7 (0.0, 8.9)	1	1.7 (0.0, 8.9)
Ecchymosis	0	0.0 (0.0, 6.0)	0	0.0 (0.0, 6.0)
Systemic reaction within 7 days	13	21.7 (12.1, 34.2)	5	8.3 (2.8, 18.4)
Fever	1	1.7 (0.0, 8.9)	1	1.7 (0.0, 8.9)
Headache	8	13.3 (5.9, 24.6)	3	5.0 (1.0, 13.9)
Malaise	8	13.3 (5.9, 24.6)	2	3.3 (0.4, 11.5)
Myalgia	6	10 (3.8, 20.5)	4	6.7 (1.8, 16.2)
Shivering	2	3.3 (0.4, 11.5)	1	1.7 (0.0, 8.9)
Reaction listed in the former Note for Guidance^b^	7	11.7 (4.8, 22.6)	2	3.3 (0.4, 11.5)
Injection-site induration ≥ 50 mm for ≥ 4 days	0	0.0 (0.0, 6.0)	0	0.0 (0.0, 6.0)
Injection-site ecchymosis within 3 days	0	0.0 (0.0, 6.0)	0	0.0 (0.0, 6.0)
Temperature > 38.0°C for ≥ 1 day	0	0.0 (0.0, 6.0)	1	1.7 (0.0, 8.9)
Malaise within 3 days	7	11.7 (4.8, 22.6)	2	3.3 (0.4, 11.5)
Shivering within 3 days	2	3.3 (0.4, 11.5)	1	1.7 (0.0, 8.9)

Abbreviations: AE, adverse event; CI, confidence interval

a Adverse events were assessed according to the International Conference on Harmonisation E2A Guideline for Clinical Safety Data Management: Definitions and Standards for Expedited Reporting.^11^

b Note for Guidance on Harmonisation of Requirements for Influenza Vaccines (CPMP/BWP/214/96)^4^

In conclusion, this study showed that the 2015 Southern Hemisphere formulation of IIV4 was highly immunogenic and well tolerated in adults, which agrees with the previous studies of IIV4 in US adults^2^ and children.^3^ IIV4 also met the requirements for influenza vaccine immunogenicity and safety.

## Supplementary Material

Supplemental_Material.docx
