# Supplementary material for: Immunogenicity and safety of the 2015 Southern Hemisphere formulation of a split-virion inactivated quadrivalent vaccine
Source: Hum Vaccin Immunother. 2017 Oct 30;14(3):593–5. doi: 10.1080/21645515.2017.1377378 (PMC5861779; doi:10.1080/21645515.2017.1377378)
Supplement: Supplemental_Material.docx [file khvi-14-03-1377378-s001.docx]

## **Supplemental Online Information for “Immunogenicity and safety of the 2015 Southern Hemisphere formulation of a split-virion inactivated quadrivalent vaccine” by Montalban et al.**

## Exclusion criteria

Potential participants were excluded if they had a history of serious adverse reaction to any influenza vaccine; had received any vaccine within 30 days before receiving the study vaccine, or plans to receive another vaccine before the end of the study; had thrombocytopenia; had known systemic hypersensitivity or a life-threatening reaction to the vaccine or any components of the vaccine; had received immune globulins, blood, or blood-derived products in the past 3 months; had a bleeding disorder or received anticoagulants in the past 3 weeks; had a history of Guillain-Barré syndrome; were seropositive for human immunodeficiency virus, hepatitis B, or hepatitis C; had a known or suspected congenital or acquired immunodeficiency; had received immunosuppressive therapy; had a moderate or severe acute illness or infection or a febrile illness (temperature ≥ 38°C) on the day of vaccination; or had any condition or illness that would pose a health risk to the participant if enrolled or could interfere with the evaluation of the vaccine. Women were excluded if they were pregnant, lactating, or of childbearing potential and not using an effective method of contraception at least 4 weeks prior to vaccination and until at least 3 weeks after vaccination.

## Ethics

This study protocol was reviewed by the Manila Doctors Hospital Institutional Review Board prior to the start of the trial. One protocol amendment (Protocol version 2.0 dated 09 January 2015) was issued and approved by the Manila Doctors Hospital Institutional Review Board as well as the Philippine Children’s Medical Center Hospital Institutional Review Board-Ethics Committee and the Mary Chiles General Hospital Ethics Research Board before the start of the study. The conduct of this trial was consistent with the standards established by the Declaration of Helsinki and compliant with the International Conference on Harmonisation guidelines for Good Clinical Practice as well as with all local and national regulations and directives. All participants provided written informed consent before being included in the trial.

## HAI assay

HAI antibody titers were measured at baseline (day 0) and 21 days (window, 21–28 days) after vaccination in all vaccinated subjects with data available, as described previously.[^1^](#_ENREF_1) Briefly, control and participant sera were incubated with type III neuraminidase to eliminate non-specific inhibitors, and anti-species agglutinins were adsorbed with a suspension of turkey red blood cells. Two-fold dilutions of the treated sera were incubated with a previously titrated influenza virus solution. The endpoint was the highest serum dilution resulting in complete inhibition of hemagglutination. The titer for each sample was calculated as the geometric mean of the reciprocal of the duplicate values. HAI titers under the lower limit of quantitation (10) were assigned a value of 5, and all HAI titers above the upper limit of quantitation (10,240) were assigned a value of 10,240.

**References**

1. Greenberg DP, Robertson CA, Noss MJ, Blatter MM, Biedenbender R, Decker MD. Safety and immunogenicity of a quadrivalent inactivated influenza vaccine compared to licensed trivalent inactivated influenza vaccines in adults. Vaccine 2013; 31:770-6. doi: 10.1016/j.vaccine.2012.11.074.
